# Cutting our own keys: New possibilities of neurodivergent storying in research

**DOI:** 10.1177/13623613221132107

**Published:** 2022-10-19

**Authors:** Hanna Bertilsdotter Rosqvist, Monique Botha, Kristien Hens, Sarinah O’Donoghue, Amy Pearson, Anna Stenning

**Affiliations:** 1 Södertörn University, Sweden; 2 University of Stirling, UK; 3 University of Antwerp, Belgium; 4 University of Aberdeen, UK; 5 University of Sunderland, UK; 6 University of Leeds, UK

**Keywords:** autoethnography, cross-neurotype communication, neurodivergent storying, neuromixed academia, non-autistic-storying

## Abstract

Increasingly, neurodivergent people are sharing their own narratives and conducting their own research. Prominent individuals have integrated the ‘nothing about us without us’ slogan, used by neurodivergent and other disabled social activists, into academia. This article imagines a neuromixed academia. We consider how to work through challenges present in neuromixed encounters; to support cross-neurotype communication and pave the way for an ethos of community and collaboration. We explore how we might create a space in which neurodivergent experiences are seen as just one part of our complex and multifaceted identities. We do this through the process of ‘cutting our own keys’, to try out new possibilities of neurodivergent storying aimed at finding ourselves in our own stories about neurodivergence. This involves borrowing and developing methodological approaches formulated outside of research on different forms of neurodivergence, and to invent our own concepts based on our own embodied experiences and the social worlds we inhabit. Throughout, we mingle our own autoethnographic accounts in relation to research accounts and theories, as a way of illustrating the work with the text as a thinking about neurodivergence with each other in itself.

**Lay abstract:**

A lot of people who do research are also neurodivergent (such as being autistic or attention deficit hyperactivity disorder), but neurodivergent people do not always feel welcome in research spaces which are often shaped around neurotypical people. Some neurotypical researchers lack confidence in talking to neurodivergent people, and others feel like neurodivergent people might not be able to do good research about other people who are like them without being biased. We think it is important that all researchers are able to work well together, regardless of whether they are neurotypical, autistic, or attention deficit hyperactivity disorder (or any other neurotype) – in truly ‘neurodiverse’ teams. In this article we talk about how to create better spaces for all researchers, where we feel valued for who we are and take each others’ needs into account. We do this using some approaches from other areas of research and talking about how they relate to our personal experiences of being neurodivergent researchers with our own personal stories. This article adds to a growing work on how we can work with people who are different from us, in more respectful and kind ways.

## Introduction

My own story feels like it is constantly in the process of being (re)written. When I spoke at the NNHMR [Northern Network of Medical Humanities Research] panel with you all, it was the first time I had been fully ‘out’ beyond the spaces that I had felt were safe. Being in a space with other people who took me as I am, showed me what I was looking for. I kept dipping my toes into my own identity, and withdrawing, afraid of being invalidated. The words ‘I’m autistic’ felt strange in my mouth, like I wasn’t allowed to utter them until a clinician confirmed what I already know, gave me the keys to my own front door. And now, it rolls off my tongue with more ease. I’ve cut my own keys, and I think I’m home. (One of us)

Reflecting on my decision to study autism as an autistic person, I sometimes think it was idealistic at best, and masochistic at worst. It’s not that I don’t enjoy working in the field, or that I believe that autistic perspectives shouldn’t be central to autism studies, because I do. Rather, it is because I made the decision to return to university for a PhD, despite being aware of the hostilities and difficulties I would inevitably face for being autistic in an overwhelmingly neurotypical space. I had sought a formal autism diagnosis to curtail future mental health difficulties, and to ensure that I would be better supported during my time at university, but this was not to be. The institution I was attending at the time decided that I was not autistic and so any accessibility requests I made would be vetoed. (One of us)

To be a neurodivergent^1^ researcher researching neurodivergence within an academic environment means working out how to form a sense of self in a cognitivist and positivist tradition mired in pathological narratives and deficit models (Botha, 2021). Entrenched within academia are presumptions about what autism ‘is’ or ‘means’, deriving from dominant discourses, which state that ‘mindblindness’ (Baron-Cohen et al., 2001), antisociality (Chevallier et al., 2012) and lack of creativity are intrinsic to autistic embodiment (Baron-Cohen & Craig, 1999). Viewed predominantly through a developmental lens in psychology, autism is regarded as an innate deficit in ability and willingness to engage in the social world, without reference to the ways in which culture or language mediates any individual’s relationship to others (Evans, 2017). This leaves little room for neurodivergent researchers to contribute to knowledge on neurodivergence or other aspects of health, identity or human experience, or to narrate their own commitments and values outside of a deficits-based model. Therefore, many neurodivergent people are excluded from disciplines that claim to represent and support them, or which defer to psychological constructs even when otherwise recognising cultural constraints on subjectivity. The neurodivergent people who remain in academia must dutifully ‘get on with things’, even when it causes burnout or distress.

Increasingly, neurodivergent people are sharing their own narratives and conducting their own research. Prominent individuals have integrated the ‘nothing about us without us’ slogan, used by neurodivergent and other disabled social activists, into academia. With the idea that ‘stakeholders’ must have a say in research concerning them, and with the neurodiversity movement thriving, it may seem that we can move onto other issues. However, there is still much to be achieved, particularly when considering the *culture* of academia. While interlinked, there is considerable difference between culture and practice, and academic culture is almost exclusively neuronormative despite the ‘interest-driven’ (c.f. D. Murray, 2018) work that attracts many neurodivergent scholars.

While neurodiversity studies (Bertilsdotter Rosqvist, Stenning, & Chown, 2020) promote a better understanding of autism and other forms of neurodivergence, these practices nevertheless belong to particular or specialised domains with little uptake within other disciplines, except in tokenistic ways (Milton, 2019). Neurodiversity studies are seldom taken seriously neither in the humanities (‘what it means to be human’) nor in medicine (dominated by deficit constructions of neurodivergence), due to presumptions that neurodivergence is somehow outside of what it means to be human (in a social or psychological sense) (Stenning, 2020), and because it does not fit neatly (if at all) into the predominant deficit construction. This is reflected in funding approaches and allocations where research on different forms of neurodivergence is monopolised by medical and educational ‘experts’ interested in what the conditions reveal about generalised human ill health and neurotypical development *in comparison* to ‘atypical’ or ‘delayed’ development (Pellicano et al., 2013). As such, neurodivergence is not considered important or interesting in and of itself, but is significant only when measured against cognitive normate human health and development. We argue that neurodiversity studies are a bridge par excellence between these fields.

This article imagines a neuromixed academia. It considers how to integrate experiences of neurodivergence into wider academic discourses, transgressing binary divisions according to received diagnostic labelling, and to enable humanity to thrive in all its multiplicity (Chapman, 2019). We want to ‘stay with the trouble’ (Haraway, 2016) of neuromixed encounters; supporting cross-neurotype communication (Hillary, 2020), paving an ethos of community and collaboration, despite the challenges arising from this. But we also want to nurture this alternative academia, allowing it not just to exist, but to flourish. We want to create a space in which neurodivergent experiences are seen as part of our identities, even if ‘neurology’ is just one aspect of our personhood. In this article we start ‘cutting our own keys’, to try out new possibilities of neurodivergent storying aimed at finding ourselves in our own stories about neurodivergence. This involves borrowing and developing methodological approaches formulated outside of research on different forms of neurodivergence, furnishing our academic kitchens for new ways of home cooking, and growing our own concepts based on our own embodied experiences and the social worlds we inhabit. The article consists of three different sections, all being part of the same kitchen, organised under the theme of *thinking about neurodivergence with each other*. We set up the structure of this new kitchen and new methods or ‘cookbooks’ for neuromixed collaboration what feminist researchers Francis and Hey (2009) have referred to as ‘joint action’. Throughout, we mingle our own autoethnographical accounts in relation to research accounts and theories, as a way of illustrating the work with the text as a thinking about neurodivergence with each other in itself. The quotes that appear in the article started as comments or discussions with each other in the development of this article, and are used to illustrate key points.

### Community participation

The research is neurodivergent-led. The whole research group is ‘neuromixed’, consisting of researchers with different neurodivergent identities, albeit dominated by autistic identities. The research questions, theories used and ways of writing, are informed by the authors’ own engagements within neurodivergent communities, thinking about neurodivergence with each other.

### The problem of storying neurodivergent selves in research and clinical practice

This, my body, this was autism – and suddenly, with the neuropsychologist’s signature on my diagnostic papers, I was no longer my body’s author. (Yergeau, 2018, p. 1)

Autistic rhetorician M. Remi Yergeau writes that, ‘[t]hrough diagnosis, autistics are storied into autism, our bodyminds^2^ made determinable and knowable through the criteria of neurodevelopmental disability’. Yergeau continues: ‘[t]hrough diagnosis, nonautistic stakeholders become authorized as “autism something” – as autism parents, as autism researchers [. . .]’ This comment reflects the prevailing cultural discourses and practices concerning neurodivergence, as those in positions of authority dictate how neurodivergent experiences are expressed, both on micro and macro levels. However, more optimistically, Yergeau adds that, ‘those who have been storied likewise respond, albeit in sometimes unexpected ways’ (Yergeau, 2018, p. 2). Within the existing cognitivist model of psychological capacities, autism is a condition that renders a subject unintelligible to themselves, since they/we cannot develop the intersubjective awareness that would allow them/us to register their/our difference. At the same time, however, autistic self-stories are reinterpreted in cognitivist narratology as manifestations of symptoms, as though linguistic meaning can be reduced to the firing of synapses rather than the available network of stories, scripts and schema. But in autism research, the stories have become naturalised and gain the appearance of inevitability. Autistic storytelling is commonly depicted as a form of ‘hacking’ or falling short of cognitive normate storytelling (see Happé, 1991); thus, any agency on the part of autistic individuals is mistakenly attributed to the autistic brain (Hollin, 2017). However, autistic people report that the greatest challenges they face are not in specific areas of ‘cognitive functioning’ but in contributing to what counts as being a worthwhile human subject. This means that a specific aspect of relational autonomy – the socially afforded capacity to ‘self-authorise’ (McKenzie, 2021) or act in accordance with our own sense of the meaning of our lives – is routinely denied to autistic people in virtue of the supposedly superior knowledge of non-autistics.

Since agency is relational rather than a matter of individual willpower, improvements in agency for neurodivergent people needs structural change. Such change should recognise the social context in which stories about neurodivergence are produced or elided (Catala et al., 2021). Commonly, ‘firsthand accounts’ within autism research are at the same time framed as authentic, more ‘experienced-based’ narratives of autism in comparison with professional or parental reports, but also dubbed ‘naïve’ or needing reinterpretation – they are, in essence, to be rewritten. Autistic voices are presented through citations – often with a sense of autistic quirkiness to stress the ‘unique vulnerabilities’ of autistic communication and the authenticity of the narrative, whereas in reality all narrators are ‘powerful’ or ‘vulnerable’ according to the context in which we communicate. Furthermore, the quest for authenticity does not consider how we are all constrained in the stories we can tell, by the subordinating/empowering labels through which we are recognised as social selves (Lucas, 2016). The problem is that individual accounts are not recognised as stories about autism because individual autistics are either ‘too autistic’ to be heard or ‘not autistic enough’ to have anything to tell (Botha, 2021):

Throughout my whole life and now into my career I have been in a paradox controlled by how I am seen by [predominantly] non-autistic people around me. They reframe and impose themselves on my narratives, stories, and self with what they believe should be [im]possible for me, in ways that retain their access to knowledge creation, but take mine. When I am speaking, thriving, or growing I am not autistic enough to be reliable or ‘right’, but when I am burnt out, floundering, or drowning, and cannot speak or act within the narrow demands of normative life, I am appropriated as evidence of the necessity of a medicalised autism. I am either unreliable or incapable, and there is always justification for the imposition of someone else’s story onto me, as if I am whiteboard, and incapable of writing my own. (One of us)

Another problem of firsthand accounts can be linked to the problem of the ‘sole autistic’ account of autism. In relation to being autistic in a shared autistic space, Sinclair (2010) has formulated the notion of ‘being autistic in one’s own space’. Bertilsdotter Rosqvist, Örulv, et al. (2020) have noted the limitations of neurodivergent individuals working more separately in their own space, in comparison with working as a neurodivergent collective, working together in a shared neurodivergent space (Bertilsdotter Rosqvist, Örulv, et al., 2020), noting how narratives of sole autistic selves tended to replicate deficit stories. It was not until writing in a collective form in a neurodivergent shared space, alternative narratives of neurodivergence started to emerge (Bertilsdotter Rosqvist, Örulv, et al., 2020, see also Jackson-Perry et al., 2020). Similarly, Bertilsdotter Rosqvist and Jackson-Perry (2021) observed how certain spaces predominantly occupied by autistic people may still be dominated by ‘non-autistic storying’ of autistic experiences, as found in a study of narratives of sexuality on an online forum for autistic people. The authors asked how we can ‘separate a person’s ‘lived experience’ from that same person’s ‘epistemic infection’ by a body of (collective) knowledge that defines their actions as being the product of deficit?’ This ‘epistemic infection’ occurs when stories that naturalise neurocognitive privilege are internalised by cognitive minorities themselves: among them neurodivergent people, who ‘buy into and even tell majoritarian stories’. Here, neurodivergent people’s own ‘non-autistic storying’ can be seen as an example of ‘minority majoritarian storytelling’ (Solórzano & Yosso, 2002), compounded by lack of access to non-pathologising concepts and language with which to shape their narratives (Fricker, 2007).

### Pointing out the impact of positionality and setting for knowledge production

Neurodivergent collective storying can be seen as a response to ‘the neurotypical gaze’ (McDermott, 2022). As counter-narratives, they can be seen as ‘critical reinterpretations of dominant narrative models’ (Meretoja, 2020). Counter-storying, we suggest, can enable neurodivergent people to leave the deficit discourse, to demonstrate narrative agency and to ‘unlearn’ the clinician’s gaze as conditioned by both space and context-specific sociality:^3^

What is the clinician’s gaze here? Why does it preclude narrative agency? A helpful clinician could allow us to display narrative agency within the process of receiving an autism diagnosis, by emphasizing the importance of our own sense of our identity [which will include recognition of relative strengths and weaknesses] rather than suggesting that we should abandon our existing desires, knowledge and commitments. Instead, autism is often presented as a monolithic identity, as a unique feature of a sovereign self that is defined as falling short of a hypothetical ideal. (One of us)

Sinclair (2010) refers to an autistic togetherness as the experience of autistic people being together in an autistic space ‘where there [a]re no NT people arranging the environment or setting the agenda’ (Williams, 1994 cited in Sinclair, 2010). This togetherness can be either verbal (based on oral or written speech) or sensorial, what can be referred as sharing an experience – sometimes within the same ‘flow state’ (McDonnell & Milton, 2014) or a sense of a collective monotropic^4^ flow (Jackson-Perry et al., 2020; D. Murray, 2018). Gemma Williams (2021) has explored the importance of being in a ‘shared cognitive environment’ as theorised by communication theorists Sperber and Wilson (1986: 41, referred in Williams, 2021, p. 126). For Sperber and Wilson this includes both ‘shar[ing] [a] physical environment’ and having ‘similar cognitive abilities’, but we also want to stress that the shared cognitive environment does not have to be ‘physical’ but, rather, an environment which is as appropriate as possible for involved interlocutors. Another form of collective storytelling is found in various autistic-authored anthologies which showcase the creative, inventive, and disruptive force of writing together. Personal essays, articles, poems, short stories, drawings and photographs collated in such anthologies (e.g. Brown et al., 2016; Huxley-Jones, 2020; Peña, 2019; Sequenzia & Grace, 2015) challenge dominant paradigms about autism and other forms of neurodivergence, providing alternative accounts and/or histories of the neurodiversity movement from those directly involved, and incorporating voices and perspectives that might not otherwise be shared (Abram, 2020). Examples include Sinclair’s essays on autistic community-building – both virtually and in person – beginning in the 1990s (Sinclair in Bascom, 2012); experiential and narrative accounts of autism and race (Brown et al., 2016); and critical responses to the medical paradigm by nonspeaking authors and activists (Peña, 2019; Sequenzia & Grace, 2015). These foreground storytelling as a subversive force both on the level of discourse (i.e. the content of the works), and also structurally (i.e. the mechanisms involved in producing these narratives).

In terms of this collaboration, our shared cognitive environment has been neurodivergent (although not exclusively autistic or similarly situated autistic), textual (as in emails and cumulative textual work on the text) and oral (regularly digitally meeting up in Zoom). However, creating an environment in which cross-neurotype communication can occur does not mean excluding those who identify as ‘neurotypical’ – nor does it mean that neurominorities are the only ones who are qualified to talk about academic barriers. What matters here is an attitude of openness to experiences that may be fundamentally different from our own, regardless of neurotype.

Working together, we have aimed at developing a shared language. The matter of a shared language is in line with what Kourti (2021) refers to as ‘more philosophically credible’ autism knowledge, or what Milton (2014a) refers to as autism research which can claim ‘epistemological integrity’. A knowledge that is created from our interactions with each other, within a neurodivergent togetherness depending on space (Bertilsdotter Rosqvist, Örulv, et al., 2020; Sinclair, 2010; Williams, 2021). This relates to the poet Joanne Limburg’s (2021) idea of the importance of thinking ‘from’ and ‘to’ autism rather than seeing it as something to do work ‘on’ (p. 19). While this may manifest differently in different disciplines, these ideas help us to think through the function of autism stories: whether they illuminate or dull our awareness of experiences that are different from our own, including those relating to harms of which we will otherwise be unaware. Interpreting our experiences through a shared lens is not to suggest that we are a homogeneous group (c.f. Sinclair, 2010). Rather than assuming that neurodivergence names specific modes of neurological embodiment, we can identify overlaps within a broader ‘neurodivergent culture’ with stories/languages, ways of reading and engaging with others, and metaphors, which in turn interact with more localised environments:

Perhaps, this is a necessary first step for ‘autistic becoming’/presence in the real world? Thinking about autism with each other – means not only talking within same neurotype collectives, but also a relational investigation in autism, an autism [or any form of neurodivergence] an ‘in and of itself’ rather than the divergence point, as considered ‘in relation to’. (One of us)

Interpreting our experiences through a shared lens encompasses a cross-neurotype collaborative autism research within neuromixed academic spaces (Bertilsdotter Rosqvist et al., 2019) and cross/interdisciplinary autism research as well. For this to work, we must develop *cross-neurotype translation practices*, working against translation practices rendering neurodivergent experiences ‘untellable’ and made to fit (neuronormative) ‘narrative scripts of disability’ (Bergenmar, 2016). Following Spivak (1993) in the context of translation studies, translation is necessary even as it remains impossible, it is a ‘friendly learning by taking a distance’ (p. 222). By translation we mean there is a need not only to engage in reflexive (friendly) learning, listening to and acknowledging difference between neurotypes, but also to learn to communicate across neurotypes differently. A reflexive attitude may fall short if it sees ‘translation’ as subsuming someone else’s response to our own discursive framework. This requires awareness that this communication is always a translation between different languages of neurotypes (different ways of communicating, socialising and processing) and acknowledging that translations are always broken and incomplete (c.f. Hillary, 2020; Milton, 2012).

We have tried to narrate ourselves in a mother tongue of another neurotype, transforming our prediagnostic mindbodily experiences into a sorted and structural diagnostic narrative. The non-neurodivergent clinical translation process contrasts with an alternative translation process, within the space of an enabling community of care, where the neurodivergent self-narrative is being produced in a space with adaptations to ‘talk with each other’. This space allows for the ‘expressive freedom’ (Crerar, 2016) that will allow us to make sense of our experiences to ourselves, and without fear of violating communicative norms that *are themselves* constitutive of authorised identities:

Why is this ‘sorted and structural’ narrative problematic [given that all narration involves some sort of ordering for intelligibility]? We do not mean that there is ‘authentic’ self-narrative that is pre-conceptual/unstructured and always authoritative. What we say about our diagnostic stories may change according to where we are in our lives. What matters more is that we are given the chance to articulate our own sense of the meanings of autism or neurodivergence AND that the listener will not impose their own understandings unreflectively. What is this ‘enabling community of care’ and what does it do differently from trying to talk to diagnostic constructions of autism? Is this just any autistic majority space or would it need to have specific features [e.g., not assuming deficits-based understandings of experiences]? (One of us)

### Talking back and talking with: a method for cutting our own keys

The six authors of this text met through mutual friends and shared connections across different geographic borders, universities and academic disciplines. Initially, five of us met with the purpose of talking about the hidden aspects of receiving an autism diagnosis for the 2021 congress of the Northern Network of Medical Humanities Research (NNMHR). Following this, the sixth author was invited into the group to complete our collective.

After hosting the NNMHR panel together, within the unique circumstances of the COVID-19 pandemic, we found that we could freely communicate, support one another’s needs as well as those of the audience, and flourish in a neurodiverse (online) space (in Zoom). For example, the participants of the panel pre-recorded their papers and the written papers were distributed before the panel within the group, which accommodated their different processing speeds. At the same time we were actively producing an alternative academic space based on our own needs, the expected presence of a non-autistic academic space also made us collectively prepare for a neurotypical academic ‘front stage’ performance (c.f. Goffman, 1959):

Those things [neurodivergent forms of communication and sociality] are not narratives about autism. But it says something about an academic space where autistic people are the norm. Where it is expected to be clear about one’s needs, to get them acknowledged without questioning, to support each other in order to accommodate those needs. At the same time we were constantly reminding ourselves that we were going to present in an academic space, where autistic people and autistic ways of processing may not be the norm, not what is expected. Where a certain pace in the panel is expected. (One of us)

While not all of us have a formal autism diagnosis or identify as autistic, we share an interest in expanding knowledge about neurodivergence and exploring how existing work on neurodivergence impacts on the lives of neurodivergent people and their families. We wanted to create a different kind of culture to talk about these things, based on mutual recognition, respect for our standpoints, and an ethics of care. This was an intuitive, self-reflective, bottom-up ‘cosmopolitical’ (c.f Stengers, 2010) methodology based on how we all try to work with others to produce knowledge. It seemed, after the conference, that we should try to explain this in writing. To be a neurodivergent academic is to either work deliberately together to recognise the validity of different forms of perception and experience, *or* to internalise subjugating ideas about our inner lives that we find also deeply problematic when applied to another minority group.

Only from the first position can we begin to consider what would count as freedom from the dual constraints of culturally normative cognitive characteristics *and* cultural stereotypes about neurodivergence. This meant questioning the ways we used available technology and giving each other the chance to question the purpose of the collaboration. One of us wrote about her experience during the preparation for the panel, acknowledging a dilemma of following her own way of functioning (detail-oriented, time for processing, monotropic, interest-based, c.f. D. Murray, 2018) at the same time articulating this a ‘problem’ in relation to what she felt was expected of her (a polytropic mind, moving effortlessly between and ‘pulling in multiple strands of information, both external and internal’ F. Murray, 2019). This illustrates an internal dialogue between non-autistic-storying and counter-storying:

What is the task actually at hand? Not what I was supposed to be doing. But what I think is most interesting. To be that autistic scholar, get inspired by other people’s ideas, follow the flow state, share the autistic togetherness through the ideas, the interest-driven hunger for knowledge, following my current interest in autistic knowledge production – its possible methods and theories, enabling contexts for autistic knowledge production. Working with some of the details, working bottom-up, from the details in your talks I found most interesting to think about, knitting them together into a sketchy, first version of a whole, a bigger picture. Taking some breaks now and then, to digest, to process, acknowledging the work I do, when I put this broader picture together into a comprehensive story. (One of us)

The cultural construction of autism as deficits in social reciprocity, or attention deficit hyperactivity disorder (ADHD) as simply a ‘learning disability’ makes it harder to recognise and articulate our own desires and commitments outside of these authorised scripts, the non-autistic-storying of ourselves, the familiar anxiety of ‘doing it wrong’ in relation to neurotypical (or more polytrophic) ways of processing, communication and sociality. So, instead of asking for individual accommodations that would aim towards a hypothetical cognitive centre, we decided to create an expansive space that could allow us all to participate as equals with different verbal/processing and attentional capacities. This led us to wonder: is it possible to embody this ethos, not only for a few hours or during discussions on neurodivergence, but in academia at large? If so, what opportunities for learning, growing, and being would this afford?

The experience of writing in a bottom-up manner, taking an interest driven approach is new to me. As someone who is often lacking in a ‘linear’ narrative, whose writing can veer off in unknown directions that make sense to me, but ‘lack clarity’ to others, I am mindful of going off on a tangent. My own writing has been critiqued for a lack of structure-something which I have worked hard to mitigate. I came to think there was a ‘right way’ of writing, and that deviation from this was likely to result in me ‘doing it wrong’. This process has not only been one of trying different ways of writing, but also has shown me what a caring and compassionate approach to working with others can look like. A space in which we don’t need to be scared of ‘making mistakes’ but are free to explore. This is funnily enough, something that I already employ with my own students [spending time during the semester where learning is interest driven, bottom up, and they can play with ideas and different forms of communication], but for some reason I hadn’t considered that this care could be extended to myself. (One of us)

The first step of this methodology is *to talk with each other*. This means to leave the ‘non-autistic space’ of the deficit discourse and return to our ‘own front doors’, talking with our own neurodivergent neighbours, from our own standpoints. This means challenging the idea of unmediated ‘firsthand accounts’ extracted from us from those who are experts into the de-contextualised meanings of our lives, instead supporting our individual and collective interpretative agency. Entering the space of our own homes, also helped us to talk from a standpoint of our own, since we were not confronted with additional sensory, attentional and emotional labour. We have realised the need for ‘cutting our own keys’, but, in order to do so, we need to work together on those keys. This is informed by our different and similar bodyminds, disciplinary and theoretical backgrounds. Part of the methodology was to develop the ideas in the text through a collective storytelling process where we have mixed personal reflections with theories from our different disciplinary and epistemological backgrounds. In line with the storytelling process as outlined by Jackson-Perry et al. (2020) in the context of sensory experience, as a form of data collection and analysis:

Broadly, this involved an iterative process whereby one of the authors started writing a free text, with no ‘guiding’ beyond ‘sensory experience’. In this way we sought to enable the person to ‘travel’ where they wanted around the theme rather than walk a path determined by pre-existing ideas of the group. [. . .] This form of data production was then carried out by the other contributing authors, initially producing three texts. These texts were circulated, added to, and commented upon by other authors during ‘virtual’ group writing rounds. [. . .] The creation and analysis of data thus became a form of intertextual intimacy. In this an ongoing dialogue between author-analysers led both to an iterative development of ‘writing up results’, and a development and refining of sensory stories – and their analysis – throughout the process.

Although the result of this process was still a linear, coherent text, allowing for a bottom-up writing approach provided a more liberating way of working for the authors, allowing them to explore writing in a more fragmented manner.

The next step of this methodology is *to talk back to power*. Talking back to power stems from a long tradition within feminist activism and critical race theory which has, more recently, been imported as a methodological approach within neurodiversity studies (Bertilsdotter Rosqvist, Stenning, & Chown, 2020). Drawing upon feminist epistemologies, we feel ‘morally obliged to speak and voice an alternative position’ even in the event that what we say is ‘not heard by all or understood or indeed deemed legitimate’ (Francis & Hey, 2009).

To talk back to power, is first, to ‘stay with the trouble’ (Haraway, 2016). By this we mean we try to work with and join teams, rather than turning away from mainstream science and cooperation between different neurotypes. Second, we ‘trouble’ the premises of the logic of deficit arguments, which maintain the subordinated positions of knower/subject and hide structural inequalities that limit our sense of collective becoming and possibility. Third, talking back to power also includes the point of talking with a collective voice, to ‘join teams’ in a shared endeavour. ‘Joint action’ is ‘core to feminist action over the years’ but in particular within academia, where we counter-narrate the position of ‘individual experts’. (Francis & Hey, 2009) Through joint action, we form a collective narrative agency. This enables plural voices and many counter-narratives. It supports one anothers’ contributions. Thus, building a collective position, a mutually supportive strategy in facilitating individual narrative agencies, we want to open up narratives about inequalities between neurotypes. We aim at a ‘discursive shift, by articulating and hence keeping alive a counter-narrative’ (Francis & Hey, 2009), carving out new positions for autistic and non-autistic researchers alike. This means choosing a way other than putting our heads down and dutifully getting on with things, according to conventional epistemic norms. This means not to withdraw, afraid of being invalidated or waiting for our experiences to be confirmed by non-autistic others. It means not trying to cope in overwhelmingly inaccessible spaces rather than discovering what it means to thrive on our own terms. It means not feeling the need to justify the point of our work except to those who hold themselves accountable for us in supportive ways. It means not becoming experts at passing. There is considerable risk in ‘cutting your own keys’ as academia is an institution that is built on hierarchical power differences, and the legacy of non-autistic narration of autistic lives has limited space for autistic people in autism research when they challenge the status-quo (Botha, 2021). Disruption is action which often comes with cost, but especially for those already on precarious contracts who do not have a permanent or secure place in academia:

Our places are often more guaranteed when we buy into the story others have written for us – each disruption I take part in, facilitate, or make becomes another hurdle I know I will probably have to overcome. I am often left choosing between creating disruption, and finding the security which I need professionally, for my personal life to be secure or to flourish. It’s another form of inter-connectivity between professional and personal for me. As such, I think it’s important we acknowledge that cutting our own keys is risky and often punished. In mixed neurotype spaces we need people with more stability and power to pave the way for those who have less, and in return for those who are slowly gaining security, to *keep* the door open, once it is our turn. (One of us)

## Using my power to speak/where do we go from here?

Narrative agency is about acquiring the power to speak as an individual, not as a ‘subject’ of dominant discourses. A first step towards this is to work towards epistemic justice (Fricker, 2007) and to create language and concepts that articulate the shared interests and harms to which we are subject as members of a group. We believe that this allows for not only a more just, but also, in line with ideas from standpoint epistemology, a more theoretically and methodologically rigorous autism research (c.f. Kourti, 2021; Milton, 2014a). The second step is that ‘found communities’ (Lindemann Nelson, 2001) afford the opportunity for recognition of our epistemic and narrative agency, even while it is questioned in mainstream culture. The process of working together has enhanced our own understandings of what feels wrong with having to participate in academic conferences to be seen as ‘doing a good job’, in particular for those among us for whom spoken language comes second to written language. Writing this together has helped us to imagine how things could be otherwise. For example, it means enlisting help to do things differently and working with people with different kinds of strengths.

We might ask ourselves, what is happening with non-neurodivergent narrative agency as neurodivergent narrative agency changes? What does it mean to support the development of another’s narrative agency? What does it mean to be in the process of formulating new words and concepts in relation to the already pre-existing words and concepts which have dehumanised and derided neurodivergent experience, new words and concepts which do not yet have a form for sharing? To start naming, making or using those new concepts, when you are so used to using the old, established ones? New metaphors and concepts invite us to inhabit a shared cognitive environment, where we can both (neurodivergent and non-neurodivergent, or neurodivergent and differently situated neurodivergent) gaze on new understandings of a similarity or difference we had not noticed before. Importantly, these shared concepts and metaphors emerge as jointly constituted concepts which evolve in and out of usefulness and clarity, sometimes taking longer to develop.

## Concluding remarks

Our understanding of ourselves and others is constantly evolving, as we share knowledge from across our different disciplines, and our individual narratives. Here we question the focus on individualistic knowledge production, which is baked into our academic systems, where the principal investigator, the first author, the lab leader is seen as the ideal position to create from. What we are doing here, and that others are doing when collaborating and sharing our space, is shaking off those normative standards and creating our own sort of thinking spaces, our own sort of academia. This is paving the way for a future where individual needs and differences aren’t pathologised, or even accommodated, but are just simply part of how we work.

We can find resonance and acknowledge difference, coming to each other as humans. Perhaps this is one of the crucial things to come from thinking about neurodivergence together – when we can be explicit about our needs, and can share space as equals, it becomes less about ‘us and them’ and more about collective authenticity. Here we align with Milton’s (2014b) idea of ‘dispositional diversity’, which seeks to emancipate all those who deviate from the narrow definitions of normality that we currently exist within. We do not need to share a neurotype to understand each other, we just have to be willing to learn from each other. Perhaps the key factor here is not even neurotype at all, but that shared desire and willingness to deviate from the prescribed norm and to create those new spaces. This is about mutual recognition. It is important that we are able to do this without sanitising our stories, or needing to make them palatable for the outsider. It is boring, unauthentic and not true acceptance. It is more masking dressed up as an attempt to convince others that we too, are human enough. Humans are messy, complex, chaotic systems, and to even begin to understand them we need to cross disciplinary boundaries, and neurological trenches to create better knowledge that serves us all.

During the work with this text, we have asked ourselves whether it is possible to shrug off neuronormative conventions, to find new ways of working and creating together in neuromixed spaces. We have explored how we might philosophically position ourselves in order to attempt this feat, and discussed of how our narrative agency might provide a new lens through which to story ourselves and others. What may the future bring, as we actively work towards an epistemological shift? Until we reach a time where neuromixed spaces and ethics of care (Laugier, 2015) are embedded into our practice, those of us who feel safe/empowered to act as academic diplomats can act as a ‘go-between’ (Stengers, 2010). Few of us openly autistic are in secure academic positions in neurodivergence-related fields. Compare that with the number of non-neurodivergent people who are working on research related to neurodivergence. What does this say about the aims of the research? This is one of the reasons why we are writing this – to provide foundations for those of us who are able to work in more ‘emancipated’ times.

## Notes

1. By neurodivergent we refer to ‘having a mind that functions in ways which diverge significantly from the dominant societal standards of “normal”’ (Walker, 2014).

2. Margaret Price’s (2015) definition of bodyminds: ‘According to this approach, because mental and physical processes not only affect each other but also give rise to each other – that is, because they tend to act as one, even though they are conventionally understood as two – it makes more sense to refer to them together, in a single term’ (p. 269).

3. We mean by narrative agency ‘the constant capacity to introduce new meaning into the constellation of relationships into which the subject is thrown’, which requires ’recognition of the subject’s speech and action’ (Lucas, 2016, pp. 10–30). This agency resides in all subjects, regardless of their perceived identities and actual support needs, as we emerge as subjects through action within a ’plurality’ (pp. 142–150).

4. Fergus Murray (2019) summarises monotropism as follows: ‘In a nutshell, monotropism is the tendency for our interests to pull us in more strongly than most people. It rests on a model of the mind as an ‘interest system’: we are all interested in many things, and our interests help direct our attention. Different interests are salient at different times. In a monotropic mind, fewer interests tend to be aroused at any time, and they attract more of our processing resources, making it harder to deal with things outside of our current attention tunnel’. The monotrophic mind is contrasted to polytropic minds, summarised by Murray as ‘have multiple interests aroused at any time, pulling in multiple strands of information, both external and internal. They are primed to be on the look-out for things like social implications, and effortlessly decode metaphors and indirect language’.
